# The New York Institute of Stomatology

**Published:** 1898-03

**Authors:** 

**Affiliations:** The New York Institute of Stomatology


### THE NEW YORK INSTITUTE OF STOMATOLOGY.

A regular meeting of the Institute was held at the residence of Dr. J. Morgan Howe, 58 West Forty-seventh Street, Tuesday evening, January 4, 1898, the President, Dr. E. A. Bogue, in the chair.

The minutes of the previous meeting were read and approved.

### COMMUNICATIONS ON THEORY AND PRACTICE.

*Dr. J. G. Palmer.*—I have the models here of the mouth of a boy between thirteen and fourteen years of age. He has five lower incisors. The occlusion is as perfect as any I have ever seen.

I would like to have those who take the trouble to look it over express an opinion as to whether it would be wise to extract one of the five incisors. The lower jaw is a little crowded, one of the incisors being twisted somewhat upon its axis and the two canines a little out of position; but the occlusion is so perfect, the general appearance so good, and the boy just at a period when he is developing into a large man, with ample room for every tooth, except the slightly contracted condition referred to, that I am at a loss to know what to advise. I brought it partly for the abnormality, partly for any information I might gain, and to put it among the other models of the Institute.

*The President.*—Our fellow-member from Paris, France, Dr. Brigiotti, has handed me two models, which he requests me to present to the Institute. One is the model of a case where the wisdom-tooth impinges upon the twelfth-year molar very extensively, and has caused probably a solution of the cementum and dentine, to the exposure of the pulp. The two teeth were extracted to rid the patient of severe pain. Dr. Brigiotti had these two teeth duplicated in hard rubber, so that the condition might be seen. The pulp is fully exposed in the posterior root. The other model which he sends contains four bicuspids on each side below and two or three above.

Dr. Geer, from Norwalk, Connecticut, has extracted a twin tooth, which he presents to the Institute. It will be seen that there are two complete teeth in the single specimen.

While the specimens are being passed around, I would like the members to take into consideration the various names which I will present to act upon the standing committees for the coming year. I believe it was suggested that each member of the committee should bring in whatever of interest might come to his knowledge, even if he were not able to consult the chairman of the committee.

*Dr. W. St. George Elliott.*—I would like to report something that has interested me of late. I have been making some tests in regard to the comparative efficiency of some of the instruments furnished us. Dentists have so much to do and so little time, that they generally accept anything that is given to them without question. There is such a thing as a poor instrument, and yet a poorer one, and the most expensive are not always the best. I took up three or four of the chip-blowers and tested them, and the tests were a revelation to me. The most expensive chip-blowers are decidedly the worst. With the most important one—the largest—I succeeded in evaporating between four and five milligrammes of water. With an instrument I made myself (it was nothing but a copper tube), I succeeded in evaporating seven milligrammes of water. The smallest and cheapest, as well as one of the newest, evaporated sixteen milligrammes of water,—three or four times as efficient as the instruments in common use.

*Dr. S. C. G. Watkins.*—I wish to say in regard to Dr. Palmer's model that we ought to leave well enough alone. The occlusion is fairly good and the appearance not at all bad. It is in the mouth of a boy. Were it in the mouth of a girl, I should perhaps consider extracting, for the sake of reducing the lower jaw, for in a girl the heavy lower jaw is always objectionable. But in the mouth of a boy I do not consider it objectionable; in fact, it may give a degree of character to the face which it would not have otherwise.

*The President.*—I do not know how often impacted teeth have tried the patience of others, but I was very glad to get the specimens which Dr. Brigiotti has sent to us, as I have had a great deal of trouble in this way. In fact, two patients were given up to die as the outcome of impacted lower wisdom-teeth, but did not. A

third case was that of a physician who came to me, saying that Dr. Hasbrouck had refused to take out his wisdom-tooth, as he was afraid of a fracture. I put a tape between that tooth and the next one, increasing the thickness of my wedge day by day until I had ten or twelve thickness of tape between the teeth, when I sent him back to Dr. Hasbrouck, who finally extracted the tooth, with very slight fracture of the lower jaw. Until this case of Dr. Brigiotti's, I never before saw a pulp of the twelfth-year molar fully exposed by the action of an impacted wisdom-tooth.

If there are no other incidents of office practice, we will close that order of business and listen to the paper of the evening by Dr. Bull, whom I have the pleasure of presenting to you.

*Dr. Charles Stedman Bull.*—First of all, an apology for a written paper instead of a spoken one; and, secondly, an additional apology, and a request for your patience in listening to a paper in which it has been impossible to exclude absolutely technical phrases.

The paper of the evening was then read by Dr. Charles Stedman Bull, entitled "The Connection between Diseases of the Eye and Diseases of the Teeth."

(For Dr. Bull's paper, see page 137.)

DISCUSSION.

*Dr. George S. Allan.*—When I called upon Dr. Bull and presented the idea to him of reading us a paper on the connection between diseases of the teeth and diseases of the eye, I may have misunderstood the doctor, but I gathered the impression that he did not think there was very much substance in the subject. So far as I know, nothing like this full and complete presentation of it is to be found; it is worthy of all credit, and certainly marks somewhat of an era in our literature.

I doubt whether many dentists are familiar with these manifestations that are constantly occurring. Certainly the dentists that I have talked with have shown very little more knowledge of the subject than I possessed myself. That we will now pay more attention to these lesions or these close connective phenomena of the organs of the teeth and the eye, I think goes without question. In my own practice I have had very few cases where I noticed any positive connection between diseases of the teeth and troubles of the eye. The most marked one I had was of a girl of twelve or thirteen, where, with very little pain, the orbit of the right eye gradually twisted around, so that in the course of three or four days the axes of the two eyes were at right angles to each other.

This condition remained unchanged for some time, until an oculist, quite a noted man, examined the teeth. Evidently his attention had not been drawn to the connection between the diseases of the eye and diseases of the teeth to such a degree as to examine the teeth in the beginning. It was only when the case proved obstinate, and he could not accomplish any good results, that he finally examined the teeth. The sixth-year molar was badly decayed and ulcerated in one of the roots. The extraction of that tooth resulted, in the course of three or four days, in a cure. The eye turned round to its normal position, and there was no further pain or trouble.

I, for one, feel much indebted to Dr. Bull for this very complete presentation of the subject, much more complete than I had expected or even hoped for, and so I have risen to start the discussion.

I would like to ask Dr. Bull if in his practice he has ever noticed any cases such as he describes where the beginning of the trouble was in the eye, and the teeth afterwards were affected by reflex action or in any way.

*Dr. Bull.*—Indirectly, yes. I have one notable case in my experience, of a man, forty-eight years old, who went to his dentist complaining of a severe pain in the second upper molar on the right side. Upon examination it was found to be a perfectly sound tooth. The pain finally left the second molar and went to another tooth, and then to the lower jaw, but always kept on the right side. Nothing wrong could be found in any of the teeth of which he complained at various times. This persisted for a number of months, to such a degree that he was forced to take narcotics. He began to use cocaine hypodermically, and then commenced to complain of flashes of light in his right eye. Some one suggested that perhaps he needed his glasses changed, as he had worn the same ones for several years. He came to me simply because certain members of his family had been to me. He said that, when looking at a light, he saw colored rings around it. Seeing rings of colored light is one of the first symptoms of the disease that we call glaucoma. This man had developed acute glaucoma, and, so far as we could determine, it had already existed about eight months. The vision remained impaired.

*The President.*—Was there any more pain?

*Dr. Bull.*—When the glaucoma became fully developed the pain became continuous, and he had excruciating pain in the eye until after the operation. It then ceased and did not return.

*The President.*—Were the teeth not diseased in any way?

*Dr. Bull.*—The dentist informed me that they were not. Several of the teeth that he complained of were filled, and it would seem probable, inasmuch as the pain in the teeth ceased with the development of the glaucoma, that it was reflex, and not due to existing disease in the teeth.

*Dr. Allan.*—It certainly seems reasonable that such conditions should exist, and that a great many of the possible cases of neuralgia, the origin of which has seemed to us very obscure, may have had their origin in some troubles of the eye, just as troubles in the eye proceed from primary troubles in the teeth.

*Dr. Bull.*—I consider it perfectly possible.

*Dr. Allan.*—Certainly, as dentists, we are constantly having cases where we feel unable to locate the primary cause, and I have known of several cases where sound teeth have been extracted without good results.

*Dr. C. A. Woodward.*—I remember a case that came to my notice, of a lady who had a great deal of trouble with one of her eyes. She had been to many oculists for treatment, without relief. The trouble extended over some years. Finally, she came to me, and I thought I detected a slight sensitiveness in one of the canine teeth which had been filled. I removed the filling, which had been near the pulp, but the pulp was still alive. I began to treat the tooth for its sensitiveness, and finally made up my mind to remove the pulp. Upon getting into the pulp-chamber I found a pulp-stone, which nearly filled it, and when that was removed the eye ceased to give trouble.

*Dr. C. O. Kimball.*—I am very sorry that I have no clinical observations to give on this matter. My attention was called to it twenty-five years ago, but it so happens that I have never had any of these cases where the eye has been directly involved in trouble beginning in the teeth. Within the last week, however, I have had a case which has bothered me somewhat, and I would like the opinion of some of the gentlemen of the Institute. A lady has been troubled with a dry joint of the lower jaw on the right side, giving pain and annoyance, and this has continued for some six or eight months. Recently she came to this city, and her physician sent her to me to see if there was any trouble with the teeth. She has not only had pain in the joint and a creaking sound on motion, but also some neuralgic pains, the cause of which I have been unable to locate. I made a very careful examination of the teeth on that side, which are more or less diseased, but found no evidence of difficulty with them severe enough to warrant the belief that the failure of the synovial fluid came from irritation of the teeth. I wish to ask if any of the other members of the Institute have seen anything which would lead them to think that this disease in the joint might have been directly or indirectly caused by trouble in the teeth?

*Dr. Benjamin Lord.*—We should all certainly feel much indebted to Dr. Bull for the elaborate paper he has favored us with this evening. He has shown that any diseased condition of the eye may be much more due to some irritation of the teeth than is generally supposed.

The subject is one that should not only interest the specialist, but the general practitioner, who, as we well understand from our experience and observation, almost entirely overlooks the influence of the teeth in a diseased condition upon the general health.

When the time comes, and it will come, I trust, that all dentists as well as oculists and aurists will study and become well grounded in general medicine and surgery, so as to understand the systemic as well as the local effects of diseased conditions of the teeth, it will tend greatly to enhance our usefulness to our patients.

I think we may consider that at least most of the cases the essayist has used in illustration of his subject had not been treated by skilful dentists, or perhaps not treated at all.

*Dr. A. G. Weed.*—I have frequently found pulp-stones the cause of pain in superior maxilla, patient complaining of pain on the floor of orbit, decided lachrymation on same side. Two such cases have been entirely cured by removal of pulp-stones from first molar. In a third case, with like symptoms, the pulp-stone was found in the first bicuspid.

*Dr. F. Milton Smith.*—I have been greatly delighted with the paper. It seems to me that, in our desire to save teeth and in our thought of the teeth, we often lose sight of those causes that are attributed to them, yet for which they are not responsible.

We ought to save every tooth that we possibly can, providing that in the saving we do no greater injury to another part of the system than that we seek to relieve. In a number of the cases that Dr. Bull has referred to, he has suggested that the extraction of the tooth has relieved the trouble. There is a tendency sometimes to resort to extraction long before it ought to be. There are many cases of diseased antrum which affect the eye. Many of these cases may be, and are, cured, and the teeth saved by proper treatment. It is a mistake to remove the tooth in these cases. I have had no extended experience with troubles involving the eyes resulting from teeth, but it would seem to me reasonable that the eye would be one of the organs affected. We do know that the teeth often are the cause of troubles with other portions of the body, the opposite being also true. I cannot discuss the paper from a scientific standpoint, but think Dr. Bull should have a vote of thanks for his very able and interesting paper.

*The President.*—I was hoping, gentlemen of the Institute, before putting this vote of thanks, which I am very glad to do, to say a word or two and to hear Dr. Bull's reply, if we might, as our evening is not quite gone yet. I was very much struck with one peculiar feature which seems to have been developed by Dr. Bull's excellent paper,—that in all the cases which he enumerated not one has been brought forward that has not been preceded by long-continued neglect of teeth. There is no instance that he mentions (Dr. Bull indicates that I am correct), where these eye difficulties or orbital difficulties supervened, in which great neglect of the teeth has not first been manifested. I did not know but that he was going to put exophthalmic goitre on the shoulders of the dental organs before he got through. In one of the instances Dr. Bull mentioned, there was an upper molar whose root was exposed, roughened, sensitive, and abscessed. Now, that to us is an impossibility, and it shows that the observer, whoever he may be (I understand Dr. Bull quoted it), was evidently not posted in the beginnings of dental disease. This paper shows me more than ever the necessity of the studies which we have undertaken to carry on for ourselves, and possibly for some others, that may eventually bring us a little nearer to the great body of practitioners of the healing art.

The instance that Dr. Bull gave us, of extracting a tooth and finding a wooden toothpick as the origin probably of suppuration which had extended very widely, is only confirmatory of what has just been stated. There was a foreign body which nature was endeavoring to expel, and it would be quite immaterial whether that foreign body were a toothpick or a tooth which had been roughened, or anything else. We must all recognize reflex action. We recognize how little children eat indigestible food of some kind or another, and through the pneumogastric nerves there is a discharge of mucus all along the track of the mucous membrane until the lungs become involved and false croup results. There is an instance of reflex action which is patent to all of us, and those of us who have brought up children probably know more acutely about it than the younger members who have not. But it seems to me that there is considerable point in a great many of the thoughts that Dr. Bull has brought up. I hope, before we pass our vote of thanks, that he will enlighten us upon some of them.

*Dr. Bull.*—In going over the literature of this subject in ophthalmological text-books and journals, the fact that impressed me more than anything else was that a description of the diseased tooth in every single case was in the language of the oculist and not in that of the dentist; and it is scarcely necessary to remark that the oculist is absolutely incompetent to make any statement as regards the existing conditions of a tooth.

The main object I had in writing the paper was to show my belief that there is no such thing as a pure, narrow, rigidly bound specialty in medicine; that every single branch of medicine is more or less intimately connected one with the other, and in talking over this subject with my fellow-oculists in this city I was surprised to find how little interest many of them took in the subject.

Now, I am a specialist in medicine, and I have always said, from the time I began as a teacher, twenty-five years ago, here in New York, until now,—and I repeated my remark this afternoon at my lecture,—that I have no belief in a pure specialist who knows nothing else. He does not even know his specialty if he does not know anything of general practice. A poor specialist in medicine, outside of dentistry, is the man who has not been a general practitioner first, and if this paper does anything towards bringing into more close connection dentistry and ophthalmology, I shall be satisfied that I have done a good thing.

A vote of thanks was tendered Dr. Bull for his valuable paper.

Adjourned.

S. E. Davenport, D.D.S., M.D.S.,
*Editor The New York Institute of Stomatology.*

